# Sleep of Children

**Published:** 1876-04

**Authors:** 


### SLEEP OF CHILDREN.

Many a bright and beautiful child is destroyed or made idiotic for life by their nurses, in one of two ways.

By the administration of laudanum, paregoric, opium, or other form of anodyne.

By teaching self-abuse, in order that the exhaustion it produces should promote sleep.

Medical books abound in cases of this lamentable character. How to guard against them with most efficacy, is worthy of inquiry.

All children, under five years of age, will be made the better, healthier, happier, and more good natured, by an undisturbed sleep of one or two hours in the forenoon.

Children, under eighteen months, may require two day naps in summer time.

If a child is regularly put to sleep at the same time, for only three or four days in succession, the habit will so rapidly grow upon it, that with the aid of quiet, and a little darkening of the room, it will, if well, fall to sleep within a few minutes of the time, for weeks and months in succession—such is Nature's love for system and regularity.

We appeal, then, to every mother, as she values the security, the health, happiness, and sanity of her children, to adopt this inflexible rule. Never allow a child to be put to sleep by any servant, on any pretence whatever, nor permit it to go to sleep at any other than the regular time; and then put the child to sleep yourself, and, if properly managed, all that you have to do is, to take the child to a quiet, darkened room, place it in the bed, with a few affectionate words, uttered in a kindly tone, leave it, and it will be asleep in five minutes, without rocking, singing, coaxing, or anything else.

It is wonderful how soon a child learns to do a thing as a matter of course, when it is put in a proper habit by a quiet and kindly firmness.

By such a plan of operation, it will be seen that all inducement to make a child sleepy, by either of the fearful practices named, is taken away from the servant. To all mothers we say, you cannot safely trust your children out of your sight with one servant in a million, and, least of all, to one of the plausible sort, who have a ready "O yes, ma'am," to every inquiry or request you have to make.

